# The role(s) of NF-Y in development and differentiation

**DOI:** 10.1038/s41418-024-01388-1

**Published:** 2024-09-26

**Authors:** Diletta Dolfini, Carol Imbriano, Roberto Mantovani

**Affiliations:** 1 Dipartimento di Bioscienze, Università degli Studi di Milano, Milano, Italy; 2 Dipartimento di Scienze della Vita, Università di Modena e Reggio Emilia, Modena, Italy

**Keywords:** Development, Gene regulation, Epigenetics

## Abstract

NF-Y is a conserved sequence-specific trimeric Transcription Factor -TF- binding to the CCAAT element. We review here the role(s) in development, from pre-implantation embryo to terminally differentiated tissues, by rationalizing and commenting on genetic, genomic, epigenetic and biochemical studies. This effort brings to light the impact of NF-YA isoforms on stemness and differentiation, as well as binding to distal *vs* promoter proximal sites and connections with selected TFs.

## Facts

- NF-Y is formed by three subunits: NF-YB/NF-YC have histone-like structures, NF-YA confers sequence-specificity.
- NF-Y is among a handful sequence-specific TFs involved in determining selection of the Transcriptional Start Sites(s) in targeted promoters.
- The NF-Y regulome is, at least in part, known, by location and functional inactivation experiments: genes involved in cell-cycle, metabolism and transcriptional regulation are among its favored targets.
- The Q-rich Transcriptional Activation Domains of NF-YA and NF-YC are alternative spliced, with isoforms showing tissue/cell-specific preference.

## Open questions

- When is NF-Y expression established during the early stages of development and does it play an important role?
- Moving forward from mESCs/ICM, is NF-Y regulated during formation of the three germ layers, and what is its impact in tissues and organ physiology?
- Is NF-Y involved in the process of cellular reprogramming and generation of induced pluripotent stem cells (iPSs) from post-mitotic -or highly differentiated- cells?
- What is the role of NF-YA isoforms splicing in these processes?

## Introduction

The CCAAT box is one of the first DNA motifs discovered in promoters of eukaryotic genes; it is typically located at −60/−100 from Transcriptional Start Sites -TSS (Reviewed by ref. [1]). It is important for promoter function, as assessed in functional experiments by mutation of CCAAT, the use of an NF-YA dominant negative mutant [1] or RNAi of the subunits (Reviewed by ref. [2]). NF-Y maintains the upstream border of the core promoter free of nucleosomes, and its removal causes relocation of TSS and appearance of extended aberrantly initiated transcripts [3]. Recent systematic studies aimed at determining enriched motifs in core promoters (−100/+100 from TSS) of coding and non-coding genes identified a very limited number of matrices, among which the CCAAT box [4–7]. In one of these studies, predictions of changes in TSS selection upon removal of CCAAT based on the AI-driven tool Puffin were validated by RNAi of NF-YA in wet experiments [5]. In initial studies on the CCAAT box, multiple proteins were shown to bind and regulate the element, notably NF-Y, C/EBP (CCAAT Enhancer Binding Protein) and NFI/CTF (CCAAT Transcription Factor) [8]. C/EBPs and NFIs turned out to bind as homo- and hetero-dimers to palindromic sites, whose biochemically characterized logos - RTTGCGYAAY for C/EBPs [9], TTGGCANNNN(G/T)CCA(G/A) for NFIs [10]- were later confirmed in ChIP-seq studies (see Factorbook.org [11]). Atomic resolution structures explain the specificity of the bZIP C/EBPs (PDB 2E43, 1NWQ, 8K8A, 1GU5, 7L4V), whereas for NFI(X) only 3D structures without DNA are available (PDB 7QQD, 7QQE).

The TF mostly associated to the CCAAT pentanucleotide is NF-Y (CBF), a heterotrimer formed by the Histone Fold Domain -HFD- NF-YB/NF-YC and the sequence-specific NF-YA subunits. Following an initial biochemical -on the TF- and genetic -on promoters- analysis, the precise CCAAT/NF-Y logo was formalized [12], inserted in TRANSFAC and JASPAR databases [13, 14] and, following validation by ChIP-seq, in FactorBook and HOCOMOCO [11, 15]. The asymmetric site extends beyond the core pentanucleotide -RRCCAATSR- with high information content. The DNA-binding details of the yeast, mammals and plant NF-Y complexes are known [16–18]. The HFD dimer contacts the phosphate backbone of the DNA in a way that is almost identical to the H2A/H2B heterodimer within nucleosomes, through basic residues of the L1 and L2 loops and α1 helix. Specificity for CCAAT is provided by the NF-YA A2 helix and GXGGRF motif. Overall, there are >35 contacts covering some 30 bps, with two important features: (i) sequence-specific contacts are in the minor groove of the DNA, leaving the major groove, typically the recognition site for most TFs, open for contacts of other factors; (ii) the DNA is severely bent -some 80°- which could also impact on the association of neighboring TFs. In essence, whenever this precise CCAAT logo is identified in regulatory regions, as in the studies mentioned in this review, one can reasonably point to the NF-Y trimer as acting on this element.

NF-YA has two major alternatively spliced isoforms -“short” NF-YAs and “long” NF-YAl- differing in 28 amino acids coded by exon-3 [19]. NF-YC also has multiple isoforms, because of two promoters and alternative splicing generated at the C-terminal [20]. In both subunits, this heterogeneity involves the Trans-Activation Domains (TADs). The NF-Y “regulome” is being understood through ChIP-Seq experiments and profiling/RNA-seq after inactivation of the single subunits.

The last 15 years have seen an exponential growth of NGS (Next Generation Sequencing) data. In addition to review and comment on experiments that focus directly on NF-Y, we add information gathered by mining this type of experiments. The first -and for the time being only- complete NF-Y knock-out mouse was reported on NF-YA, yielding clear results: KO mice died *in utero* at early embryonic stages, since no homozygously deleted embryo was recovered at 8.5 dpc [21]; this posed a significant roadblock to further genetic experiments in the early phases of development, at least in mammals. Thereafter, only conditional KO models were reported, in several tissues and organs [22–27]. We will comment on them in sections devoted to the distinct systems.

mESCs are a useful model of differentiation of mesoderm, endoderm and ectoderm germ layers by formation of Embryoid Bodies in vitro [28]. Originally derived from cells of the Inner Cell Mass (ICM), they retain full capacity to generate a new organism. NF-Y is present and required for growth of mESCs, apparently partaking in the core transcriptional program with Oct4, Sox2, Klf4 and Nanog [29–33]. A genetic screen identified NF-YA among the few genes modulating Oct4 activities [34] and a proteomic study concurs that NF-YC is part of interacting complexes [35]. Through RNAi of NF-Y subunits and ChIP-seq, Jothi’s lab found robust genomic connections of NF-Y with the stem TFs, supporting the notion that it is a “pioneer” TF in mESCs [33]. The same conclusion was reached in other cell systems [36].

These results begged questions concerning the role of NF-Y in development and differentiation: some of the answers are now available -at least in part- and the studies are reviewed hereafter.

## *Ex uno, ad pauca*: from zygote to blastocyst

### Mice

The first indication on the importance of NF-Y in pre-implantation embryos came from a study in mouse [37]. The Authors mapped DNAse I-hypersensitive sites -DHS- used as a proxy for “open” regions of accessible chromatin, at the zygote, 2-, 4-, 8-cell and morula stages, to identify regulatory regions mediating Zygotic Genome Activation (ZGA), the process by which the genome of the fecundated oocyte starts to express its own genes (Reviewed by Schulz et al. [38–40]). DHS were relatively few in the zygote, some 800; they increased slightly at 2-cells −1000- and dramatically at the 4-cell −3k- and 8-cell -12k- stages. Note that the Authors did not separate early from late 2-cells. The data were matched to expression analysis and gained functional sites considered at each step; two TFs matrices were mostly enriched: (i) Oct4 in enhancers at the 8-cells stage when ZGA is widespread; (ii) NF-Y, present at 2-cells stage on promoters, together with GC-rich matrices related to the Sp/KLF (Krüppel-Like Factor) Zn-finger TFs. NF-YA was then knocked down by RNAi, showing that a sizeable number of DHS present at 2-cells were decreased/abolished. These results were later confirmed using a different approach: computing nucleosomes (re)-positioning during ZGA found a restricted number of associated TFBS, including NF-Y, catalogued as “major” drivers based on expression patterns [41]. The other matrices -Srebp, Klf6, Etv1, Usf2, Rfx1- are of TFs with which NF-Y has genomic connections, based on ENCODE ChIP-seq data, and synergy in activation [42–44] and References therein. Expression of *Nf-ya* mRNA was found in oocytes, suggesting maternal derivation in zygotes. Huang et al. developed a tool -dbEmbryo- that integrates multi-omics data from many studies on mouse pre-implantation embryos, which confirmed an open *Nf-ya* locus at 2-cells [45]. Interestingly, deposition of the H3K4me3 “positive” histone mark happens later, at 4-cells, and best correlates with gene expression at 8-cells, suggesting that the TFs bound at 2-cells function as “pioneer” for recruitment of “writers” of histone marks. Further support for a very early role of NF-Y comes from experiments on Tankyrase (TNKS), a Poly(ADP-ribosyl) polymerase that regulates β-catenin and oocyte-to-zygote transition to 2-cells. Addition of IWR1, a TNKS inhibitor, inhibits remodeling of chromatin and ATAC-seq identified enrichment of NF-Y, SPs/KLFs, and PITX1/OTX1 sites, consistent with the respective TFs being maternally expressed and playing a role in the early events of transcriptional opening [46].

### Humans

ZGA initiates later in humans, at the 8-cells stage: experiments analogous to those described above were reported using LiCAT-seq, a technique measuring at the same time chromatin accessibility and gene expression [47]: a progressive opening of sites from the zygote is shown, with major changes in accessibility at 4-cells and transcription at 8-cells, implicating a selected group of TFs. The NF-Y loci were among the most numerous already at 4-cells -and earlier- with SPs/KLFs. Indirect evidence is provided by another study showing that Differentially Expressed Genes -DEG- gained between the 4- and 8-cell stages are enriched in two terms -*mitotic cell-cycle* and *DNA metabolism*- and include genes that are known NF-Y targets [48]. These studies also confirm a previous report on activation of Transposable Elements (TEs) in ZGA, notably LTR5, LTR7 and LTR12 [49]: LTR12, specifically, is bound and regulated by NF-Y [42, 50–53].

### Bovine

ZGA in bovines is also delayed with respect to mice; ATAC-seq experiments again implicated NF-Y in the 4- to 8-cells transition, with OTX1, KLFs and, as expected, DUX4 [54]. Sites were gained earlier on promoters, apparently kept functionally inactive and then activated at ZGA. Importantly, analysis of expression indicates high levels of the HFD subunits during the process, but very low levels of NF-YA at 2- and 4-cells and activation at 8-cells, mirroring the increase of NF-Y locations. This is consistent with (i) NF-Y/CCAAT sites being apparently absent in genes active in bovine differentiated spermatozoa [55]; (ii) expression of NF-YA being absent in mouse spermatocytes, whereas that of NF-YB/NF-YC is high [56]. Thus, this is an indication that NF-YA is the limiting subunit of the trimer, required for CCAAT-binding in a physiological system. The same study also analyzed histone PTMs associated to enhancer *loci*, finding an enrichment of DUX4 at 4-cells in association to H3K27ac and H3K4me3, whereas NF-Y -and KLF4- were bound at 4-cells in loci without these histone marks [55]: this suggests that the NF-Y-KLFs/SPs combination does not require them for access to DNA. In summary, accessibility of selected TFs precedes -and potentially later induces- deposition of “epigenetic” histone PTMs. These data were confirmed and expanded by findings that NF-YA is induced by and mediates PDGFRβ signaling: an inhibitor compound impacts negatively, and an agonist positively, on embryo development and implantation potential [57].

### Frog

ZGA happens later in frog, with some 8 rounds of cell divisions preceding it. Little is known about the role of NF-Y in early maturation of amphibians, but three reports concur that the DNA-binding activity of NF-Y -identified as the complex binding in vitro to the canonical HSP70, GATA2, and MARCKS CCAAT boxes- is maternally derived, increasing following Mid-Blastula Transition -MBT- which coincides with ZGA. Notably, the chromatin-bound fraction -extracted with high salts- was responsible for the increase at MBT, whereas low-salt extractions of nuclei -which measures chromatin-unbound trimer- yielded constant amount of NF-Y [58–60].

### Zebrafish

Miao et al. generated a fish model in which Nanog, Pou5f3 and Sox19b (NPS) -the homologues of mammalian Nanog, Oct4, and Sox2- were genetically ablated: the triple mutant showed lack of ZGA, which was not achieved in single mutants [61]. Functionally, expression of about a third of genes was robustly down-regulated (Group 1), consistent with an equivalent number of active promoters losing accessibility and lacking RNA Pol II on TSS; a third of the genes were moderately affected (Group 2), and the rest were unaffected (Group 3). In these latter units, DNA matrix analysis highlighted NF-Y, Sp1, KLF9, FOXK, PATZ1, ATF1; note that Sp1 and KLF9 were more enriched in Group 2, with TBP and BBX. ChIP-seq analysis of NF-YA then confirmed equivalent binding to Group 3 locations in *wt* and NPS mutants, indicating that the NPS TFs do not recapitulate all pioneers acting at ZGA, with a sizeable number of active *loci* targeted by NF-Y.

In studies focusing on Prep1, a TF member of the TALE (Three-Amino-acid Loop Extension) family (Reviewed by Selleri et al. [62]), genomic locations were enriched in NF-Y sites: functionality and direct interactions between NF-YA/NF-YB and Prep1 were reported [63]. Incidentally, these data reinforced previous ChIP-seq data on association of related TALE TFs with NF-Y/CCAAT in human cancer cells [64] and in ENCODE datasets [44]. In a second study, the same group analyzed another TALE TF -PBX4- at ZGA (3.5hpf), using ChIP-seq and Dominant Negative technology [65]: they gathered lists of controlled genes and confirmed co-binding of PBX4 with NF-Y, with a recognizable geometry of respective sites, TALE-10bp-CCAAT. Finally, the sites proved to be functional when linked to a heterologous promoter in enhancer assays in vivo, in a PBX4/NF-Y-dependent manner. The same TALE/NF-Y geometry also emerged in experiments using mouse embryo fibroblasts -MEFs- by looking at Sp2 locations in *wt* and *sp2*^–/–^ cells: the Suske lab first identified CCAAT boxes, rather than the predicted GC-rich matrix of SPs [66, 67]; then, they pointed to the TALE-10bps-CCAAT matrix, showing that it was important for Sp2 association and function, and that Sp2 facilitates DNA-binding of TALE/NF-Y complexes [68]. Altogether, these results point at this combination as important for early embryogenesis, placing knowledge of the 3D details of this evolutionarily conserved combination high among future structural studies.

These Zebrafish studies do not bear implications as to the precise trajectories leading from zygote to ZGA, as mentioned above in mammals, but a hint comes from DANIO-CODE, a consortium that systematically catalogues gene expression, genetic and genomic data in *Danio rerio* [69]: using MARA, a tool predicting promoter function based on the presence of TFBSs, the Authors found three matrices with widespread effects on expression patterns, among which NF-Y, further predicted to have maximal function in the initial phases of embryo development, and decreasing after the sphere stage.

**Fig. 1:**
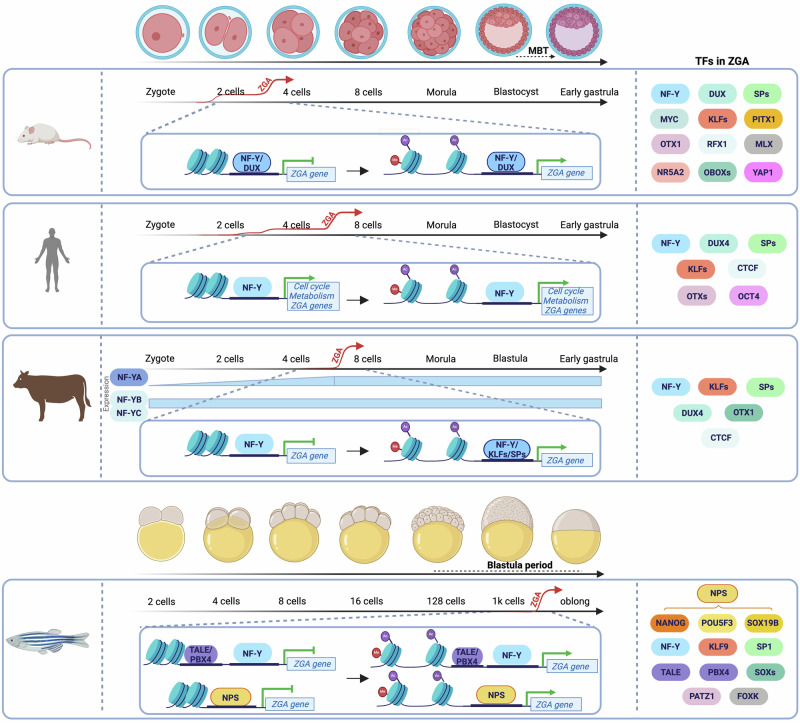
Timing of zygotic transcriptional onset and NF-Y activity in different vertebrates. The main transcription factors (TFs) participating in zygotic genome activation are indicated on the right. The transcript levels of NF-Y subunits in cow have been reported by Halstead et al. [54]. NPS = NANOG, POU5F3, SOX19B.

Overall, it is fair to conclude that (i) NF-Y is a pioneer TF acting in the early phases of ZGA with specific partner TFs; (ii) binding is -at least initially- promoter-restricted and precedes the deposition of histone PTMs typical of active chromatin (notably the most common ones, analyzed in these studies); (iii) this scenario is evolutionarily conserved, at least in vertebrates (Fig. 1).

## *Ex paucis, plures:* NF-Y in development of lineages, tissues and organs

### Male germ cells

Several reports in different species point at NF-Y/CCAAT as very important in driving expression of genes involved in differentiation of spermatozoa. The first indication came from ATAC-seq analysis in SSEA3^+^ hSSC (Human Spermatogonial Stem Cells) [70]: in addition to hormone receptors, major TFBS are NF-Y and DMRT1/6, master TFs driving sex dimorphism in general, and differentiation of spermatozoa in particular [71]. Although less precise than the TALE/NF-Y mentioned above, a clear bias in the geometry of the respective sites was found. The combination was absent in differentiating cKIT^+^ spermatogonia, and thus deemed specific for the unipotent stem cells. Additionally, open chromatin in TEs was found exclusively at LTR12 sites -LTRC/D/E- enriched in CCAAT. These data were confirmed by analysis of stem and differentiated populations of testis by scRNA-seq [72, 73]. DHS in mouse germ cells during embryogenesis have CCAAT enriched in male *vs* female primordial germ cells (PGCs): importantly, in a window of post-mitotic differentiation between d13.5 and d14.5, NF-Y sites are gained in enhancers of genes whose promoters remain repressed and marked by H3K27me3; thereafter, these units are postnatally activated and remain expressed as hallmarks of adult SSCs [74]. This finding confirms that the role of NF-Y is not restricted to being a promoter activator, but it can act at distance, and it is not necessarily associated to ongoing transcription. By analyzing male germ cells using transgenic mice in which the regulatory regions of ID4 -a marker of stemness- drive eGFP, CCAAT was found enriched in DEG of “SSC-enriched” ID4^bright^, but not of ID4^dim^ “progenitor-enriched” spermatogonia (cKIT^+^) [56]. These latter results might be -subtly- different from those mentioned above in humans [70]; note that there might be differences in species, as the TFBS reported in mouse and human PGCs open locations are rather dissimilar [74].

NF-Y is likely to play a role also at later stages of spermatogenesis: CCAAT was found in ATAC-seq sites of pachytene spermatocytes, the stage past cKIT^+^ and undergoing meiosis, but not in round spermatids [75]; in spermatids, CCAAT becomes prevalent only in DEG upon treatment with JQ1, an inhibitor of BRD4, a “reader” that recognizes acetylated histones [41]. Additional scATAC-seq studies confirmed enrichment of CCAAT in hSSCs, and in cells at late stages, Zygotene, Pachytene/Dyplotene [73], as previously shown by bioinformatic analysis of large datasets from mice [76]. Finally, transcriptomic analysis of Sea Bass gonads also suggests that *nf-y* subunits expression are associated to testis development [77].

While these data from vertebrates suggest an important NF-Y role in development of male germ cells and in the maturation of spermatozoa in adults, the point is actually proven in the invertebrate planaria *Schmidtea mediterranea. Smed-NF-YB* originally emerged from an RNAi screening searching for genes important for SSCs [78]. The Newmark’s group reported that *Smed-NF-YB* is essential in SSCs; *Smed-NF-YA1*, *Smed-NF-YB2* and *Smed-NF-YC* show lethality upon RNAi, because of defects in somatic cells, possibly ectodermal lineages [79], as also suggested by another study [80]. The data on *Smed-NF-YB* are consistent with defects in maintenance, rather than specification, of early sperm cells; differentiation is initiated normally, but the number of differentiated cells drops substantially over time, likely because of lack of proliferation of precursors [79]. In the same study, a similar phenotype was reported upon RNAi of *Sm-NF-YB* in *Schistosoma mansoni*: in this case, the gonadal phenotype was comparable to that of mice knocked out of *Taf4b* [81] or *Plzf* [82, 83]. TAF4 is an HFD subunit of the General Transcription TFIID complex, PLZF is a zinc finger repressor. Intriguingly, analysis of TAF4b ChIP-seq in developing oocytes of mice -d16.5- identified NF-Y and Sp1/KLFs as enriched motifs [84]; the same was found by CUT&RUN analysis in male spermatogonia [85]. PLZF ChIP-seq also yielded CCAAT/NF-Y as primary TFBS [86]. Overall, these data suggest that the three proteins -NF-Y/TAF4/PLZF- partake in the same SSCs transcriptional program, reinstating the importance of NF-Y in recruiting TFs and cofactors to target sites, either through direct interactions, recognition of TATA (TAF4) or yet to be determined mechanisms (PLZF).

In *C. elegans*, an early study showed that *CeNf-ya1* mutants have fewer germ cells in gonads of hermaphrodites and, as consequence, reduced capacity to generate an offspring [87]. RNAi screenings of 183 TFs in search for genes involved in differentiation of Somatic Gonadal Precursors (SGP) identified *CeNf-yb* among those whose inactivation causes appearance of hmcs (head mesodermal cells), which are markers of differentiation [88]. In mutants, the number of SGPs was lower, unlike the number of descendants at the L3 stage, or in gonads at the L4 stage: this suggests a role of *CeNf-yb* in stem cells self-renewal, not in differentiation of precursors. Overall, the *S. mediterranea*, *S. mansoni* and *C. elegans* experiments agree on a role of NF-Y subunits in determining the identity of male germ stem cells, and possibly in proliferative capacity exiting from the stem cell compartment, rather that lineage specification and differentiation.

### Hematopoiesis

The role of NF-YA in the hematopoietic stem cells (HSCs) was essentially studied by the S. Emerson lab, with overexpression -OE- and conditional KO experiments. OE of NF-YAs was performed by retroviral infection and by protein transduction -TAT-NF-YA- in primitive stem- cells [89, 90]: (i) early precursors were expanded in vitro, showing a remarkable increase in the capacity to populate -and competitively re-populate- the bone marrow of irradiated mice in vivo (Bone Marrow Transplantation, BMT); (ii) expression of stem cells genes -HoxB4/C4, Lef-1, Notch1, Hes-1- was increased; (iii) upon culturing of BMT-derived cells, primitive precursors (LSK, Lin^-/lo^/Sca-1^+^/c-Kit^+^) doubled in NF-YA OE cells, mature myeloid precursors did not change, and differentiated cells halved [90]. In the experiments with protein transductions, human CD34^+^ cells were used and CD45^+^ HSCs were recovered more efficiently, compared to controls, after BMT [89]. The NF-YA protein is normally high in B and T lymphocytes, lower in erythroblasts and myeloid cells: conditional KO in HSCs -driven by inducible *Mx1-cre*- eliminated NF-YA within 3 days, leading to a dramatic phenotype: most mice died of BM failure by d11 post-deletion in competitive BMT [91]. Indeed, the only mice surviving in noncompetitive BMT were the rare ones with incomplete *Nf-ya* deletion. Characterization of BM subtypes in vitro showed that HSCs and multipotent progenitors were unaffected in *Nf-ya* KO when kept quiescent, but dropped dramatically upon induction to proliferate. HSCs were blocked in G2/M and then underwent apoptosis: expression of stemness and G2/M genes decreased significantly, already after 24 h of deletion, inducing an unbalance of pro- and anti-apoptotic gene expression. These results are recently confirmed in studies focusing on the role of CDK6, a Cyclin-dependent kinase. First, NF-Y and SPs were initially shown to be the landing platform of CDK6-regulated genes, with both TFs being phosphorylated by CDK6 [92]; in a subsequent study, the kinase was shown to initiate a program geared to activate HSCs [93]. A kinase-dead mutant blocks NF-Y activation of these genes, switching the program toward maintenance of stemness, so the mechanism is possibly mediated by interaction with -and phosphorylation of- NF-YA [92]: the residue involved -S320- is close to the DBD, and it is known to impact on the stability of the DNA-bound complex [94]. Taken together, these observations indicate that NF-YA becomes crucial upon exit from quiescence, possibly through post-translational modification(s). Other experiments also suggested a role of NF-Y in expanding immature BM cells: overexpression of the Dominant Negative mutant YAm29, forming a trimer but preventing CCAAT-binding, yielded a reduction in the number of myeloid (CFU-M) and granulocyte (CFU-G) precursors: as in the KO study, expansion, not differentiation, of immature cells was affected, since differentiated MAC-1^+^ cells did appear, and more rapidly, in liquid growth conditions [95]. Overall, these data agree that NF-YA depletion leads to severe impairment in the proliferative potential of all lineages, but not in the initial phases of differentiation.

The mRNAs of all NF-Y subunits were shown to be expressed in circulating, non-cycling human monocytes, as were HFD proteins, but the NF-YA protein appeared only upon maturation to macrophages [96], along with antigen-presenting genes, the system in which NF-Y was originally described [97]. Additional profiling experiments in mouse BM macrophages stimulated with CSF1/LPS [98], human macrophages activated with IFN and TNF [99] and post-mitotic mouse macrophages induced to monocytes with IL4 [100] concur on the enrichment of NF-Y/CCAAT in DEGs. This was ultimately shown in a comprehensive study on DEG promoters in bone marrow-derived macrophages [101]. In reprogramming of chromatin domains -enhancers and Topologically Associated Domains- upon postmitotic differentiation of monocytes or dendritic cells, CCAAT/NF-Y was found in *loci* associated to H3K27ac upon elimination of Cohesin (RAD21) or CTCF, in which case NF-Y/CCAAT sites became prevalent [102]. Surprisingly, given its notorious crucial role in shaping Topologically Associated Domains, the effect of *Ctcf* KO was functionally minor. This result suggests that CCAAT/NF-Y -and other identified TFs- can vicariate CTCF, but not Cohesin. In granulocytes, NF-Y/E2F modules are apparently the landing matrices of CEBPe, a bZIP TF known to play a key role in terminal differentiation [103]. The study shows repression of MYC-driven G1/S transition and of G2/M promoters by CEBPe, through a mechanism independent from the repressive CHR/CDE/DREAM complex.

Expression of NF-Y subunits is also regulated during erythropoiesis, as it increases from ProCFU-E to nucleated erythroblasts (See bloodspot.eu). One study found NF-Y as the major TF localized to sites with increased accessibility upon deletion of SETD8, the methylator of H4K20me1, required for erythropoiesis [104]. This PTM is promiscuous, being associated to inactive, as well as active chromatin. At terminal stages of erythrocytes maturation, the function of NF-Y/CCAAT in the globin gene clusters has been studied for decades (Reviewed by [105]). Specifically, the γ-genes are expressed only in the fetal period: the binding of the BCL11 repressor is required to prevent NF-Y association to the double CCAAT boxes, which leads to switching to β-globin expression at birth [106, 107]; BCL11, in turn, recruits the repressive NuRD complex [108]. Single bp mutations around the distal CCAAT box of the A^γ^ promoter are associated to Hereditary Persistence of Fetal Hemoglobin (HPFH) in humans, a condition in which repression does not occur and fetal hemoglobin is maintained in adult life. Elegant experiments using CRISPR-Cas showed that different HPFH mutations prevent BCL11 binding, allowing NF-Y to pursue the activator function beyond the fetal period [109, 110]. The exception is the −113 A>G mutation relative to the γ-globin transcription start site, which prevents binding of both BCL11 and NF-Y, while creating a novel site for the erythroid TF GATA1, which activates in cooperation with the proximal NF-Y/CCAAT [109]. This system exemplifies the importance of the timing of repressor/activator access to a small area of the promoter, as well as of back-up systems in which “new” sites can vicariate lack of binding of the intended TFs; it is also notable that the proximal CCAAT is spared by mutations, possibly because of closer proximity to the core activities of the TSS.

### Skeletal muscle

Proliferating myocytes express all NF-Y subunits, but terminally differentiated myotubes, which are *syncytia* of fused, multinucleated cells, express very little, if any, NF-YA (long isoform) [111–113]. Genetic ablation of *Nf-ya* in adult muscle stem cells -termed Satellite Cells, SCs- was obtained crossing *Nf-ya*^flox/flox^ with inducible *Pax7*-CreER mice [24]. Under physiological conditions, the phenotype was apparently mild, in agreement with the almost undetectable levels of NF-YA in adult myotubes. However, a decline of SCs and of SC-derived cells in the subsequent steps of differentiation, suggests long-term effects. The picture changes upon muscle injury -by cardiotoxin- entailing regeneration of fibers since KO mice showed a slow-down in cell expansion out of the SC compartment and impairment of late stages of myotubes formation. Furthermore, analysis of DEG in SC indicates DNA-damage and pro-apoptotic signatures. The data imply a key role of NF-YA in muscle regeneration, but not in normal-adult- physiology. That NF-YA is expendable for muscle differentiation was further proven by experiments in rhabdomyosarcoma (RMS). A CRISPR-Cas9 genetic screening based on isolation of myocytes that differentiated from RMS lines identified the three NF-Y subunits among the top hits driving differentiation [114]. The mechanism lies in shut-down of CCAAT-mediated expression of the pathogenetic cause of RMS, the PAX3-FOXO1 oncogenic fusion. This work further clarifies that expression of NF-Y subunits is far from required, and indeed possibly detrimental, for terminal differentiation of myotubes.

Another TF relevant to myogenesis is Rev-Erbα, a nuclear receptor repressor whose elimination is sufficient to trigger myotubes differentiation. ChIP-seq of Rev-Erbα in murine C2C12 identified CCAAT/NF-Y, rather than the expected RORE matrix [115]. This finding is not unusual for Rev-Erbα, which binds to tissue-specific TFBS in other cell types: HNF1 in liver, C/EBP in brain, NF1 in adipose tissue [116]. Direct interactions with NF-YB were reported [115]. Importantly, MRF -Muscle Regulatory Factors- genes and cell-cycle promoters were shown to be co-regulated by NF-Y and Rev-Erbα, notably repressed *via* pausing of RNA Pol II during proliferation; Rev-Erbα is then released at the onset of differentiation, and the process is enhanced by treating cells with a Rev-Erbα antagonist. The Authors concluded that Rev-Erbα/NF-Y are involved in keeping myocytes in a proliferative status, preventing differentiation. The link is further supported by experiments in mESCs: NF-Y/CCAAT was the major TFBS in DEG of Rev-Erbα/β double KO cells [117] and, consistent with the muscle study, *cell-cycle* genes were at the top of the GO category of down-regulated genes. That MRFs are under NF-Y regulation is further shown in a study in which exon-3 of NF-YA was ablated in C2C12 cells: the NF-YAs-expressing myocytes, while growing normally and being phenotypically indistinguishable, lose differentiation capacity as they apparently lack muscle commitment [118].

Additional data came from analysis of muscles of individuals affected by Fascioscapulohumeral Muscular Distrophy 2 (FSHD2), a human genetic disease caused by mutations in SMCHD1, a chromatin modifier. A downstream effect is overexpression in muscle precursors of DUX4, normally confined to a restricted window of ZGA: DUX4 OE indeed causes defects in differentiation of muscle cells [119]. Jiang et al. performed single nuclei RNA-seq, identifying two distinct populations based on DUX4 expression, FSHD-DUX^low^ and FSHD-DUX^high^, the latters showing low expression of differentiation genes and signatures of increased proliferation [120]: the TFBS enriched in the FSHD-DUX^high^ are E2Fs, NF-Y and FOXM1. Since DUX4 activates expression of NF-YA [121], a possible explanation of the effect on proliferation might be indirect through NF-Y.

### Cardiomyocytes

A first circumstantial indication on a role of NF-Y in heart development came from mice knocked out of *Nkx2-5*, a TF that plays an important role in heart formation, whose mutations -in humans- are associated to Congenital Heart Disease -CHD- and whose ablation in mice is embryo lethal [122]. Expression analysis in *Nkx2-5* heterozygotes at d12.5 revealed upregulated DEG enriched of CCAAT/NF-Y in promoters [123]. Subsequently, two studies by the Olson lab identified NF-YA as crucial in cardiomyocytes physiology. In the first, characterization of cellular populations in regenerative and non-regenerative mouse heart by single nucleus RNA-seq led to the definition of a cluster of cells, termed CM4, specifically linked to regeneration. It is composed of immature cardiomyocytes that proliferate after injury: the specific units were guided by NF-Y, ETS, SRF and NFE2L1 matrices [124]. These sites were also enriched in ATAC-seq datasets in the same experimental window. OE of NF-YA and SRF spurred proliferation of immature cardiomyocytes and, separately, NFE2L1 conferred pro-survival to H_2_O_2_-mediated oxidative stress. Finally, NF-YA and NFE2L1 OE induced proliferation/regeneration and, most importantly, myocardial protection from induced ischemic injury in vivo. In the second study, NF-YA was conditionally knocked out in embryonic cardiomyocytes, leading to decreased proliferation, likely because of altered expression of cell-cycle, glycolytic and OXPHOS genes [26]. In turn, this led to structural cardiac malformations, with consequent malfunction, and death of the embryo. Cardiac fibroblasts, whose proliferation following a pathologically stiff extracellular matrix is dependent on NF-YA in vitro and in vivo, contribute to myocardial stiffening and dysfunction [125]. We remark that the regenerative problems described in these mice are similar to those of skeletal muscle.

Additional data come from Zebrafish embryos. Challenged by morpholinos against NF-YA, a cardiac phenotype was manifest as proliferation of cardiomyocytes was dramatically decreased between 48 and 72 hpf [126]. This was at least partially due to lack of binding to cell-cycle genes, resulting in a reduction in the number of cells and formation of a “small” heart. Heart rates were only marginally altered, but eventually the animals did not survive past 120 hpf, likely because of loss of cardiac output. The phenotype was partially recovered by co-injection of *nf-ya* mRNA. Note that Zebrafish has two *nf-ya* genes and it is not clear which one was targeted or OE in this study.

### Smooth muscle

An in vivo study investigating NF-Y in vascular smooth muscle reported on NF-YA increased levels upon artery injury in neo-intimal lesions, in macrophages, smooth muscle and endothelial cells, leading to increased NF-Y binding to the CyclinB1 promoter and cell proliferation. PDGF-BB induces this cascade *via* AKT and ErK1/2 signaling, but the effect was eliminated -in vitro- or attenuated -in vivo- by overexpressing the NF-YA DNm29 mutant [127].

### Nervous system

Over the past decade, the Nukina group generated several conditional *Nf-ya* KO models in post-mitotic neuronal cells, initially in pyramidal, cerebellar and motor neurons [128–130]. The phenotype becomes evident over a relatively long period of time—20 to 40 weeks- with mice ultimately dying by one year of age. The pathway investigated was expression of protein chaperones controlling ER physiology. The KO mice displayed progressive cellular alterations and ER disorganization, including deposition of insoluble proteins resulting from misfolding. The role of NF-Y in activating chaperones genes that counteract ER stress has been known for decades [131], including synergy with one of the master TFs, ATF6 [132]. In a recent study, embryo neuronal cells were conditionally KO of NF-YA through crossing with *Nestin*-CRE, resulting in dramatic brain alterations and death in late embryogenesis [27]. Downregulation of cell-cycle genes was coupled to an apoptotic response. As for isoforms, expansion of neural progenitors was associated to NF-YAs, whereas NF-YAl was turned on at later stages, during differentiation. Overexpression of NF-YAl, but not NF-YAs, by *in utero* transfections yielded a decrease in proliferation of progenitors as if the former competed negatively for the same sites of pro-proliferative genes activated by the latter.

The same lab had previously characterized the role of NF-Y in Hungtington disease (HD), a genetic neurological disorder with dominant transmission, whose pathogenesis is caused by increased length of the Poly-Q tract at the N-terminal of Huntingtin (Htt). This is known to be associated with altered expression of several genes, including the HSP70 chaperon: mutant, but not normal Htt sequesters NF-YA -and NF-YC- into large aggregates in vitro and in Htt-mediated “inclusions” found in brain cells in vivo, decreasing its nuclear levels: binding to the HSP70 promoter and expression of the chaperon is consequently affected [133]. Expression profilings of normal *vs* HD brain cells concurred that NF-Y is indeed a major TF in promoters of DEG with reduced expression [134, 135]. *Nf-ya* mRNA expression was induced in late stages of the disease in HD mouse models, presumably to compensate for the decreased levels of the functional protein; indeed, this is in accordance with a negative auto-regulatory transcriptional mechanism regulating *Nf-y* genes expression [135–138].

These data point at the NF-Y/Htt connection as relevant to explain the pathogenesis of HD, a notion substantiated by results from other neurological diseases caused by the expansion of Poly-Q in different genes. In SBMA -Spinal Bulbar Muscular Atrophy- the CAG triplet expansion is found in the Androgen Receptor -AR- gene: mut-AR interacts with NF-YA, decreasing expression of TGFβRII, causing alteration of TGFβ signaling, in turn involved in the progression of the disease [139, 140]. SCA17 -Spinocerebellar Ataxia 17- is caused by Poly-Q increase in the TATA Binding Protein, TBP: two papers reported on a scenario similar to HD and SBMA, namely increased binding of mut-TBP to NF-YA, matched by decreased expression of chaperon genes -HSP70, HSPA5- which worsens the outlook of the disease by increasing the production and deposition of insoluble toxic aggregates in neurons [141, 142]. In a subsequent in vivo study, Huang et al. overexpressed TBP proteins with intermediate and large numbers of glutamines: in the former, mice showed the expected neurologic phenotype, with substantially increased mut-TBP/NF-YA association; in the latter, an additional phenotype of muscle degeneration emerged, associated to loss of mut-TBP binding to the MyoD MRF, entailing altered expression of muscle genes and loss of commitment of myoblasts [143].

Additional information came recently from studies in *C. elegans*. A genetic screening for regulators of the *flp* neuropeptide in IL1 (Inter labial neuron Type 1) neurons identified *nfya-1*, whose mutation abolishes expression in lateral and dorsal, but not ventral neurons [144]. A similar phenotype is observed in *nfya-2* mutants, and double mutations affect also IL1 ventral neurons, as do mutations in *nfyb-1* and *nfyc-1*. The Pocock lab searched genes involved in the development of glutaminergic PVQ interneurons, isolating a mutant that affects the *nfy-1c* gene. They then analyzed mutants of the other *nfy* subunits, all expressed in PVQ neurons, obtaining similar phenotypes. The Authors went on to prove that *nfya-1* controls fate of the VA motoneuron, in addition to PVQ, as well as Pan-neuronal gene expression. A set of genomic and genetic experiments identified the NFYA-1 regulome, among which prospective TFs helping NF-Y accomplish the double task of being a general, as well as a lineage-specific activator [145]. Pan-neuronal and neuron-specific expressions were deemed controlled by distinct sets of TFs and the pan-neuronal activity of *nfy* genes was not reported in the Heo et al. study, since pan-neuronal markers *rgef-1*, *unc-119* and the IL2-specific *tba-6* were not affected [144].

*C. elegans nfy* genes were also implicated in (i) expression of *egl-5*, cell-fate determining Hox TF [87]; (ii) tail morphogenesis through *tbx-2*, a master TF whose expression is required for pharyngeal muscles, repressed by *nfya-1* through CCAAT boxes: mutation in the element -or feeding animals with *nfya-1* siRNA- leads to loss of binding and ectopic expression of TBX2 in other tissues [146, 147]; (iii) vulva development [148]; (iv) dimorphism in CEPso glia cells [149]. In this latter report, *nfya-1* mutation affects adult, but not juvenile, expression of genes in hermaphrodites: by relieving repression, male-specific expression of markers is established in CEPso glia. The same happens in *nfyb-1* and *nfyc-1* mutants and the trimer acts downstream of *mab-3*, the worm homologue of the DMRT TF mentioned above. The noteworthy common theme of these transcriptional systems in *C. elegans* is a repressive role of *nfy*.

Finally, a recent manuscript describes the role of NF-Y in wound-induced tissue regeneration in *Hofstenia miamia*, specifically leading to production of neurons and, ultimately, of a “new” brain [150]. NF-Y was suspected because of the prominence of its CCAAT matrix in time-course experiments monitoring ATAC-seq *loci* during the process; thereafter, the Authors performed RNAi experiments proving that NF-Y genes -the combination of NF-YA, NF-YC, and two NF-YBs- are high in the hierarchy of TFs guiding the process. By analyzing scRNA-seq, it was concluded that NF-Y expression is high in the “progenitor” stem cells group that generates different neuronal subtypes: importantly, it drives the expression of *soxC*, a key player in early differentiation out of stemness, by binding to the promoter following regeneration. Prospectively, the description in the ATAC-seq *loci* of other matrices familiar as CCCAAT companions -bZIP, E2Fs- point the way to future experiments. Altogether, studies from *C. elegans* and *H. miamia* -and to some extent mice- are starting to delineate the network driven by NF-Y in stemness and the targeted TFs that lead to exit from it.

### Liver

The hepatic role of NF-YA was illustrated by two conditional KO mouse models. A first study reported dramatic liver injury, with ALT and AST markers increasing considerably by week 4 after deletion [151]. Histologic observations documented progressive cellular degeneration with enlarged nuclei, depletion of glycogen, lipids accumulation and cellular necrosis, resulting in inflammatory infiltrates and formation of regenerative nodules. Molecularly, the cause of the phenotype was ascribed to altered expression of ER-stress genes: the pathway is complex and potentially indirect, since the *Xbp1* and *Chop* TFs -both under NF-Y control- are upregulated in the KO mice, as well as the *Grp78* chaperone.

The second model showed modest reduction in body weight and fat, and more pronounced for glycogen [23]. In this case, it is not clear the timing and the severity of the phenotypic changes. In animals and isolated hepatocytes, the ability to produce glucose -gluconeogenesis- is affected, and this is, at least partially, due to lack of activation of specific genes, in which NF-Y teams up with CREB. Similarly, lipogenesis -both cholesterol and triglycerides- is low, because of decreased expression of known NF-Y targets *Acaca* and, more profoundly, *Fasn*. In summary, the severity of liver phenotypes are in between the relatively mild, slowly emerging defects observed in neurons/myocytes and the brutal ones in HSCs.

### Pancreatic β cells

A second example of the importance of NF-YA for cellular -and organismal-homeostasis was established by KO in pancreatic β-cells using *Ins2*-CRE [25, 152]. Decreased NF-YA levels are seen in Obese (Ob/Ob), Streptozotocin-treated and in aging mice, all conditions of Type2 Diabetes. The reduction was partial -to 34%-, likely reflected in no changes in life expectancy, adipose mass, lipids in the plasma or liver weight. However, mice had higher glucose levels, both under normal and high-fat diets, because of decreased insulin concentration; in turn, this was due to diminished β-cells islet mass and lack of expansion following high-fat diet, as in normal mice. β-cells developed normally in embryos but were not expanded postnatally and the defect was traced to lack of proliferation, rather than cell viability. GLUT-2, the sensor of glucose levels, is under NF-Y control and might be the key effector, since *Glut-2*^–/–^ mice show similar phenotypes [153]. Further experiments by crossing heterozygotes to *ob/ob* obese mice led to worsening of the β-cell phenotype, as a result of decreased antioxidant production, increased ROS and apoptosis [152]. Apparently, this was due to altered expression of the anti-apoptotic *Bcl2*, with respect to pro-apoptotic *Bax*.

### Adipocyte

Conditional *Nf-ya* KO in adipocytes represents the third system illustrating the importance of NF-Y in control of metabolic pathways. The plasma levels of the Leptin hormone correlate with the mass of adipose tissue: an increase in the latter entails higher Leptin levels in the blood, promoting a physiologic fasting response; loss of adipose mass lowers Leptin levels, altering basal metabolism and neuroendocrine signals to stimulate feeding. CCAAT/NF-Y is important for expression of *Leptin* [154]; the Friedman lab produced a conditional *Nf-ya* KO model using *Adiponectin*-CRE, showing a -modest- loss of body weight over a period of 28 weeks, but a substantial loss of fat mass [155]. This was not due to the mice becoming “lean”, but rather because of an authentic lipodystrophy, a condition due to a metabolic syndrome typically associated to decreased levels of leptin, insulin-resistant diabetes, hyperlipidemia and steatosis [156]. All parameters of the metabolic syndrome were ameliorated by treating mice with leptin, but not with a high-fat diet, proving the point that the adipose tissue degeneration, due to the *Nf-ya* KO, was causative, not an epiphenomenon, of the unbalance.

## *Ex pluribus, unum*: NF-Y in reprogramming

A circumstantial role of NF-YA in reprogramming of differentiated cells to iPS is suggested by some data, although functional experiments are missing. During late phases of reprogramming of SSCs to multipotent cells, expression of NF-YB is upregulated, being involved in sustaining the expression of stem cell maintenance genes, such as Oct4/Nanog, Sox2, as well as Fgf4 [157]. Jaber et al. reprogrammed MEFs with different combinations of TFs, to obtain pluripotent or trophoblast stem cells, and accessibility of the genome was monitored at different steps: the *loci* becoming active had enrichment of NF-Y/CCAAT, and other TFs [158]. More specifically, investigating the patterns of AS changes during reprogramming, a switch was observed from NF-YAl -in fibroblasts- to NF-YAs, in iPSC [159]. Cell reprogramming can also be obtained by Somatic Cell Nuclear Transfer (SCNT): Djekidel et al. profiled chromatin accessibility of donor cells and embryos at different stages of the process and the close-to-open *loci* important for totipotency were enriched in NF-Y/CCAAT [160]; NF-YA was found to be expressed at low levels in donor cells, increasing in one-cell SCNT embryos, thus suggesting a role of NF-YA in reprogramming.

## NF-Y splicing isoforms: tissue specificity and functional activity

**Fig. 2:**
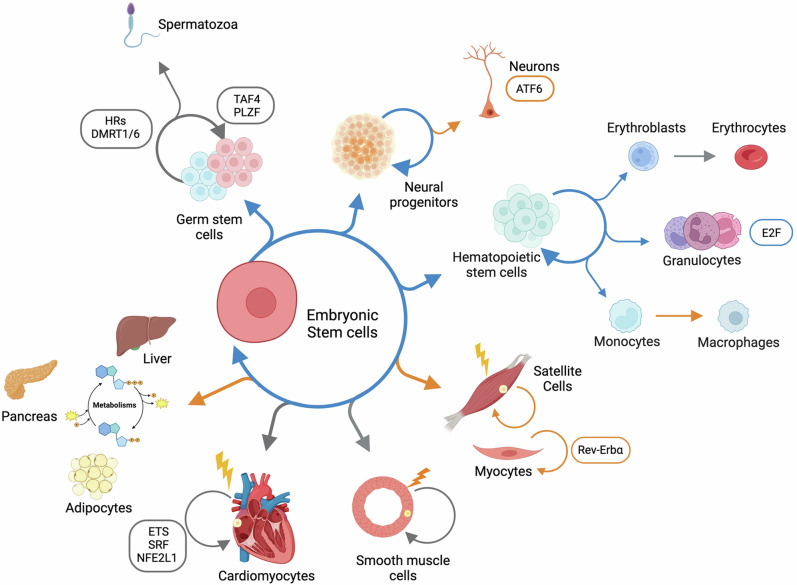
NF-Y involvement in proliferation and differentiation of different cells and tissues. Arrows indicate the NF-YA isoform involved in the represented processes: orange = NF-YAl, blue = NF-YAs and gray= not defined.

Although the presence of at least two NF-YA -and multiple NF-YC- isoforms has been known for decades, it was only recently that this aspect came under scrutiny. Unfortunately, in many functional studies reported above, it is not clear which NF-YA -or NF-YC- isoform is present in the cell line used. Moreover, in most of the OE systems reported, there was no mention as to which of the two major NF-YA isoforms was used, making interpretation of the results difficult. On the other hand, several other studies showed that alternative splicing of NF-YA is implicated in the regulation of stemness, differentiation, and cell death through tissue-specific expression and regulation of different transcriptional programs (Fig. 2). The expression of NF-YAs is dominant in thymus, spleen, bone marrow, whereas NF-YAl is the main isoform in liver, brain and lung [19, 91, 130, 161]. At the cellular level, NF-YAs is expressed in stem cell populations –mES cells and HSCs– and NF-YAl is predominant in differentiated cells –neurons, myocytes, fibroblasts. The expression of NF-YA isoforms changes throughout mouse ES differentiation: NF-YAl increases in ES cells either treated with RA or induced to form EBs, while NF-YAs significantly decreases [29, 31].

Analogous results came from mouse HSCs: NF-YAs is expressed in immature c-KIT^+^ BM cells and declines upon hematopoietic differentiation [90]. NF-YAs OE by retroviral infection or protein transduction in mouse primitive hematopoietic cells pointed at the key role in HSC self-renewal and in vivo repopulating ability through activation of transcriptional programs, among which upregulation of *Hox* genes [89, 90]. In the muscle system, the short NF-YA transcript is expressed in embryonic and fetal myoblasts together with NF-YAl. In post-natal muscles, a general drop in NF-YA expression is observed, with only NF-YAl being barely detectable [24]. Results from muscle SCs are discordant from mouse embryonic and HSCs in that they express NF-YAl, necessary for the maintenance of the adult stem cells pool, genome integrity and early differentiation. NF-YAl OE experiments and forced NF-YAs expression through CRISPR-Cas9 exon-3 deletion in immortalized myoblasts confirm different activities of the two isoforms in this system [113, 118]. As for cardiomyocytes, the important studies mentioned have not investigated expression and role of the individual isoforms.

In the nervous system, RNA-seq data from mouse brain tissues including striatum, cortex and hippocampus, highlighted expression of NF-YAl (estimated ratio of exon-3 inclusion = 0.7 ~ 1.0) [130]. Analyses on whole cortices isolated from *wt* mice at various embryonic and postnatal stages show that the predominant NF-YAs in neural progenitors is replaced by NF-YAl during differentiation, again highlighting a correlation between NF-YAs, stem/early progenitor cells and cell-cycle progression [27]. Finally, a novel NF-YA splice variant - NF-YAx - characterized by exon-3, exon-5 and partial exon-7 skipping has been described during mouse embryonic development [162]. Mouse embryo mRNAs show dominant expression of NF-YAs at stage E8.5, NF-YAl at stage E9.5-E18.5 and NF-YAx in main body from stage E12.5-14.5 embryos. Although the physiological activity is not clear, NF-YAx overexpression is cytotoxic to embryonal neural-lineage progenitors, which undergo KIF1Bβ-dependent necroptosis.

As for NF-YC, at least three splice variants are produced, which show cell-type preference, apparently reciprocal with respect to NF-YA isoforms, in that the 50 kD appears to be more abundant in lines with NF-YAs, and the smaller NF-YC 37 kD in cells with NF-YAl [20]. Although nothing has so far been reported on physiologic activities in development or differentiation, a recent study suggests a specific role of the short 37 kD in bladder cancer development [163]; note that we were unable to detect major changes in NF-YC isoforms expression -at the mRNA level- in several types of epithelial cancers [2], so this might be peculiar to bladder, a topic deserving further investigations.

## Conclusions and perspectives

The data reported are mostly related to NF-YA, reinstating its regulatory role within the trimer; they are consistent with NF-Y being crucial to provide proliferative potential to stem cells, as well as to prevent apoptosis. A potential practical way to use the current knowledge could be envisaged in tissue regeneration, for example upon injury in muscle or engraftment of HSCs, providing that efficient -and safe- delivery of the -appropriate- isoform is obtainable. A fundamental difference between promoter *vs* enhancer binding is emerging, both in terms of timings and function in early development. As to the formers, there is now sufficient evidence that NF-Y plays a pivotal role in determining TSS selection [3], along with a handful TFs, on a sizeable number of mammalian promoters [5–7]. In keeping with this, recent analysis of NF-Y binding in 16 mouse and human cell types -immortalized and transformed- as well as normal tissues seem to suggest that a large proportion of NF-Y sites -larger than previously documented- are indeed bound to promoters [164]. As to enhancers, which are apparently not primary sites along the initial phases of development, one scenario posits that NF-Y helps recruitment of cell-type specific master TFs [33]. It will be important to understand why enhancer sites which equal promoter locations in ENCODE cancer cell lines, are less prominent in normal cells. It will also be relevant to quantify and characterize binding to TEs of retroviral origin, many of which have enhancer capacity. One also wonders whether NF-Y is in reality involved in promoting transcription of eRNAs, ncRNAs originating from enhancers, which are important for *locus* activation [165], an aspect not investigated so far.

Mechanistically, sets of TF partners is emerging, whose molecular connections with the NF-Y trimer could be dissected by structural studies: SPs/KLFs, TALEs, CREB, DMRTs, for example. To this end, the recent spectacular improvement of predictive systems (AlphaFold) could allow systematization of screenings with neighboring TFs, with the resulting working models based on the atomic details of multiprotein complexes to be thereafter biochemically verified.

Finally, it is becoming clear that the two major NF-YA isoforms play different roles: how this is accomplished, by targeting different genes or recruitment of different cofactors on the same genes, is an open matter. Additional genome editing experiments aimed at exon-3 elimination could shed light on this issue.
